# Association of plasma BMP6 levels with the rates of brain atrophy in older people without dementia

**DOI:** 10.3389/fneur.2025.1559219

**Published:** 2025-07-15

**Authors:** Xin Zhang, Pan Fu, Yan Cai

**Affiliations:** Department of Neurology, Taizhou First People's Hospital, Zhejiang, China

**Keywords:** Alzheimer's disease, BMP6, MRI, brain atrophy, hippocampus

## Abstract

**Background:**

Bone morphogenetic protein 6 (BMP6) has been implicated in the pathogenesis of Alzheimer's disease (AD), and its levels have been reported to be associated with cognitive performance. However, few studies have examined the association between plasma BMP6 levels and brain atrophy in older adults.

**Methods:**

A total of 340 older adults without dementia were included in the current study. Study participants had baseline plasma BMP6 data available and at least two structural MRI scans. Volumes of six brain regions were measured, including the hippocampus, entorhinal cortex, middle temporal gyrus, fusiform gyrus, ventricles, and whole brain. A series of linear mixed-effects models were built to examine the associations of plasma BMP6 levels with brain atrophy over time.

**Results:**

Our study revealed that higher plasma BMP6 levels were associated with a reduced rate of volume loss in the hippocampus, entorhinal cortex, middle temporal gyrus, and whole brain. However, there was no significant link between plasma BMP6 levels and changes in the volume of the fusiform gyrus or ventricles.

**Conclusion:**

Our results may provide novel insights into the mechanisms of neurodegeneration in AD, contributing to new avenues for timely intervention and potentially slowing disease progression.

## Introduction

Alzheimer's disease (AD) is characterized by progressive cognitive decline and neurodegeneration and is the most common cause of dementia (1, 2). AD is associated with substantial brain atrophy, particularly in the hippocampus and entorhinal cortex, regions crucial for memory and learning (3, 4). Recently, the search for blood biomarkers that can predict or monitor disease progression and neurodegeneration has gained increasing interest (5, 6).

Bone morphogenetic protein 6 (BMP6), a member of the transforming growth factor-β superfamily, has been implicated in various biological processes, including neuronal differentiation and axonal growth (7). A previous study has shown that BMP6 levels are significantly increased in the brains of AD patients and APP transgenic mice (8). This elevation in BMP6 is accompanied by impaired hippocampal neurogenesis (8). A recent longitudinal study has examined the association between blood BMP6 levels and cognitive performance in older people (9). This study suggested that higher levels of blood BMP6 are associated with better cognitive function, highlighting the potential of BMP6 not only as a therapeutic target but also as a biomarker for early detection and monitoring of AD progression (9). However, the relationship between plasma BMP6 levels and brain atrophy, as measured by MRI, has not been previously explored.

To fill this gap, we aimed to investigate the association between plasma BMP6 levels and six MRI-based regional brain atrophy, including hippocampus, entorhinal cortex, fusiform gyrus, middle temporal gyrus, ventricles, and whole brain. Our findings could facilitate early detection of neurodegeneration in AD, allowing for timely intervention and potentially slowing disease progression.

## Methods

### Alzheimer's Disease Neuroimaging Initiative (ADNI) study

Data of the current study were downloaded from the ADNI database (adni.loni.usc.edu). The ADNI study was initiated in 2003 with the primary goal of examining whether demographic, clinical, neuropsychological, neuroimaging, and biological markers can be integrated to track the progression of mild cognitive impairment (MCI) and mild AD. The ADNI study was approved by the Institutional Review Boards of each participating center, and written informed consent was provided by each study participant. More detailed information can be found on the ADNI website (adni.loni.usc.edu) and has been described previously (10).

### Study sample

We selected study participants who had baseline plasma BMP6 data available and at least two structural MRI scans. In the current study, we included a total of 340 older adults without dementia, including 52 participants with normal cognition (NC) and 288 participants with MCI. The criteria for NC included a Mini-Mental State Examination (MMSE) (11) score between 24 and 30 and a Clinical Dementia Rating (CDR) (12) score of 0. The criteria for MCI included an MMSE score between 24 and 30, a CDR of 0.5, a subjective memory complaint, and objective memory loss as measured by education-adjusted scores on the Wechsler Memory Scale Logical Memory II, with no significant interference with daily life activities.

### Measurement of plasma BMP6 levels

Plasma BMP6 levels were examined using a multiplex-based immunoassay panel based on Luminex immunoassay technology and the detailed procedures of plasma collection and measurement have been described elsewhere (https://adni.loni.usc.edu/wp-content/uploads/2010/11/BC_Plasma_Proteomics_Data_Primer.pdf). Briefly, plasma proteins including BMP6 levels were examined using a subset of plasma samples from the ADNI cohort by a 190-analyte multiplex immunoassay panel. The panel was developed on the Luminex xMAP platform by Rules-Based Medicine (RBM) and contains multiple proteins (13). We used the quality-controlled values of plasma BMP6 in all statistical analyses. Plasma BMP6 values were expressed in ng/mL. Values were log transformed before statistical analyses.

### Measurement of MRI neuroimaging markers

The ADNI imaging procedures can be found on the website (https://adni.loni.usc.edu/data-samples/adni-data/neuroimaging/mri/) and have been described previously (14). Volumetric segmentation of T1-weighted sagittal 3D MPRAGE sequences from MRI scans were processed using FreeSurfer image analysis software (http://surfer.nmr.mgh.harvard.edu/) by the ADNI investigators at the University of California, San Francisco (15). We extracted volumes of the hippocampus, entorhinal cortex, fusiform gyrus, middle temporal gyrus, ventricles, and whole brain from the ADNIMERGE dataset. To account for brain volume differences related to head size, we calculated adjusted volumes using the following equation: Adjusted volumes = raw regional brain volume/total intracranial volume ×1,000.

### Statistical analysis

We used Pearson's correlation tests to assess the relationships between baseline plasma BMP6 levels and structural regional brain volumes of hippocampus, entorhinal cortex, middle temporal gyrus, fusiform, ventricles, and whole brain. To investigate the associations of plasma BMP6 levels with the rates of regional brain atrophy among older people without dementia, a series of linear mixed-effects models were performed, adjusting for potential covariates. A total of 6 linear mixed-effects models were built for regional brain volumes (hippocampus, entorhinal cortex, middle temporal cortex, fusiform, ventricles, and whole brain), which were treated as the dependent variables. Models included main effects of age, gender, education, APOE4 status, MMSE score, plasma BMP6, and their interactions with follow-up time (years). Each model included a random intercept for each subject. All statistical analyses were conducted using R statistical software. *P* < 0.05 was considered statistically significant.

## Results

### Baseline characteristics

In the present study, we included a total of 340 older adults without dementia [mean age, 74 [SD = 7]; 130 women [38%]; mean education, 16 [SD = 3]; 171 APOE4 carriers [50%], mean MMSE score, 27 (2)]. Table 1 summarizes demographics and imaging data of the study participants. The adjusted volumes of hippocampus, entorhinal cortex, middle temporal gyrus, fusiform, ventricles, and whole brain were 4.21 (SD = 0.73), 2.19 (SD = 0.49), 12.1 (SD = 1.62), 10.59 (SD = 1.39), 26 (SD = 13), and 643 (SD = 42), respectively. The mean of baseline plasma BMP6 was 0.86 ng/mL (SD = 0.31).

**Table 1:** Sample characteristics.

**Characteristic**	***N =* 340**
Age, years	74 (7)
Education, years	16 (3)
**Gender**	
Male	210 (62%)
Female	130 (38%)
**APOE4 status**	
APOE4 non-carriers	169 (50%)
APOE4 carriers	171 (50%)
MMSE	27 (2)
Hippocampus	4.21 (0.73)
Entorhinal cortex	2.19 (0.49)
Middle temporal gyrus	12.10 (1.62)
Fusiform	10.59 (1.39)
Ventricles	26 (13)
Missing, *n*	1
Whole brain	643 (42)
Plasma BMP6 levels, ng/mL	0.86 (0.31)

Continuous variables, such as age and education, are summarized as mean (SD). Categorical variables, such as gender and APOE4 status, are summarized as n (percentage).

APOE, Apolipoprotein E; MMSE, Mini-Mental State Examination; BMP6, Bone morphogenetic protein 6.

### Cross-sectional relationships between plasma BMP6 and demographic and cognitive variables

Spearman's correlation and two-sample *t*-tests were performed to examine the relationships between plasma BMP6 levels and demographic and cognitive variables. Plasma BMP6 levels were not associated with age (rho = 0.04, *p* = 0.43) or years of education (rho = 0.07, *p* = 0.19). No significant differences in plasma BMP6 levels were observed between males and females (*t* = 0.83, *p* = 0.41), or between APOE4 carriers and non-carriers (*t* = −0.04, *p* = 0.97). However, plasma BMP6 levels were positively associated with MMSE scores (rho = 0.11, *p* = 0.035).

### Cross-sectional relationships between plasma BMP6 and MRI neuroimaging markers

**Figure 1:**
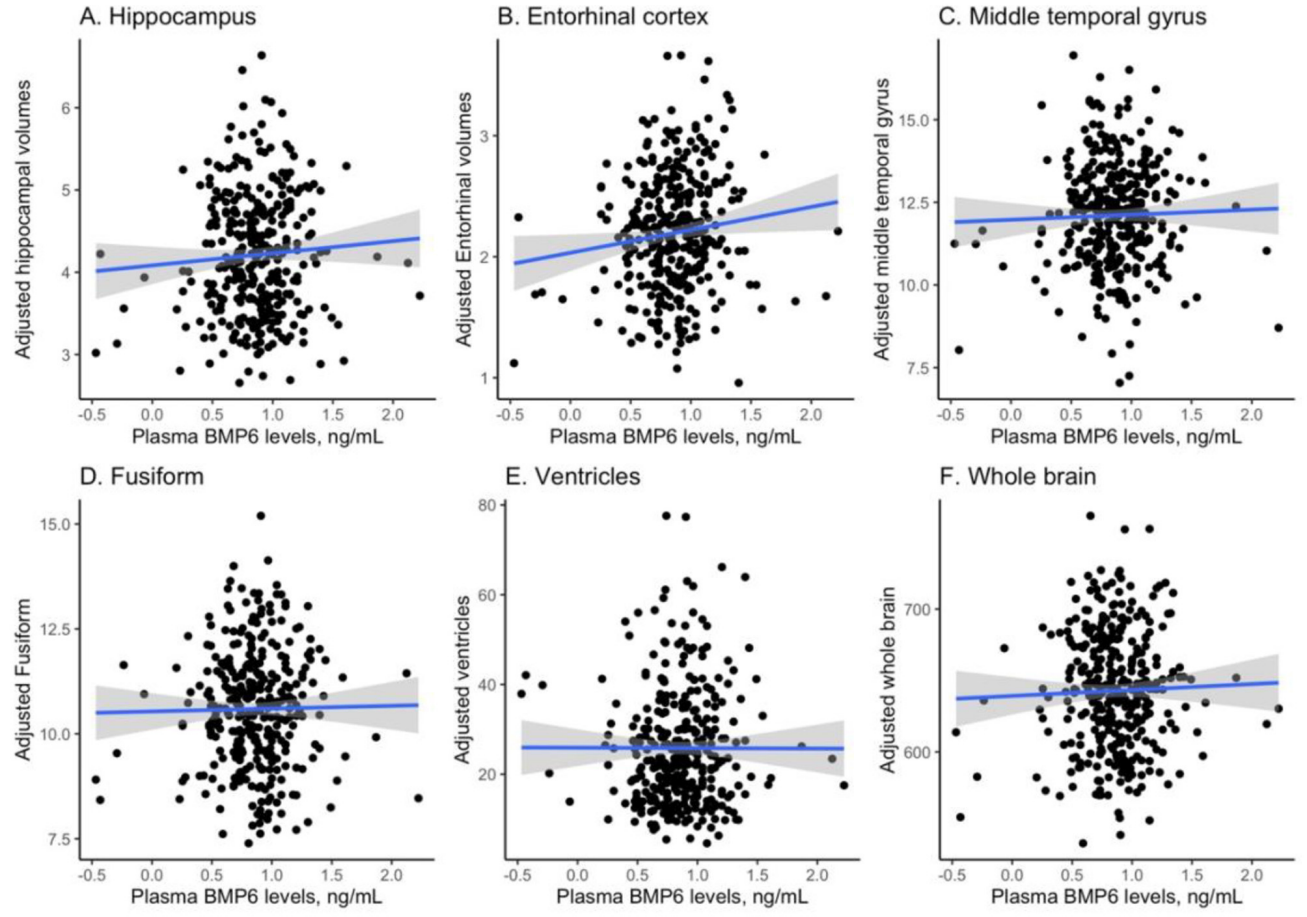
Cross-sectional relationships between plasma BMP6 levels and regional brain volumes. BMP6, Bone morphogenetic protein 6. **(A)** Hippocampus. **(B)** Entorhinal cortex. **(C)** Middle temporal gyrus. **(D)** Fusiform. **(E)** Ventricles. **(F)** Whole brain.

Pearson's correlation tests were conducted to examine the relationship between plasma BMP6 levels and 6 MRI neuroimaging markers, and Figure 1 visualizes the relationships. As displayed in Figure 1A, plasma BMP6 levels were not correlated with volumes of hippocampus among older adults without dementia (*r* = 0.06, *p* = 0.25). As shown in Figure 1B, plasma BMP6 levels were positively correlated with volumes of entorhinal cortex among older adults without dementia (*r* = 0.12, *p* = 0.026). As demonstrated in Figure 1C, plasma BMP6 levels were not associated with volumes of middle temporal gyrus among older adults without dementia (*r* = 0.03, *p* = 0.59). As displayed in Figure 1D, plasma BMP6 levels were not correlated with volumes of fusiform among older adults without dementia (*r* = 0.015, *p* = 0.78). As displayed in Figure 1E, plasma BMP6 levels were not correlated with the enlargement of ventricles among older adults without dementia (*r* = −0.002, *p* = 0.97). As displayed in Figure 1F, plasma BMP6 levels were not correlated with volumes of whole brain among older adults without dementia (*r* = 0.03, *p* = 0.57).

### Associations of plasma BMP6 levels with the rates of brain atrophy

**Figure 2:**
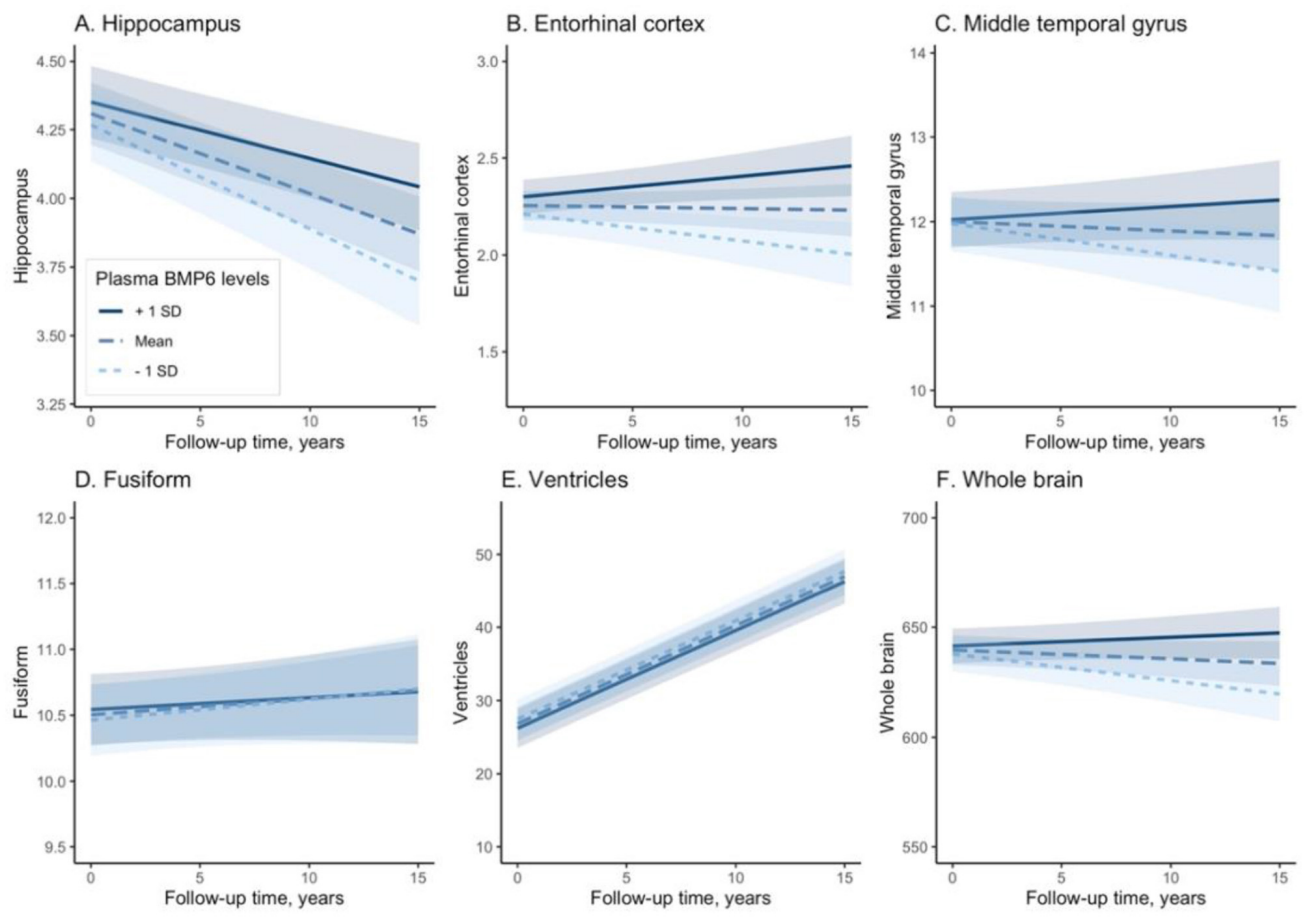
Associations of plasma BMP6 with the rates of regional brain atrophy. BMP6, Bone morphogenetic protein 6. **(A)** Hippocampus. **(B)** Entorhinal cortex. **(C)** Middle temporal gyrus. **(D)** Fusiform. **(E)** Ventricles. **(F)** Whole brain.

We built six linear mixed-effects models for each MRI neuroimaging marker, which was treated as the dependent variable in each model. Regression terms of six linear mixed-effects models were demonstrated in Table 2. In the model with hippocampus as the dependent variable, the interaction term between plasma BMP6 and years (follow-up time) was significant, indicating that higher baseline plasma BMP6 levels were associated with a slower rate of atrophy of hippocampus (coefficient [95% CIs] = 0.029 [0.016, 0.041], *p* < 0.001; Figure 2A). Likewise, in the model with entorhinal cortex as the dependent variable, the interaction term between plasma BMP6 and years was significant, indicating that higher baseline plasma BMP6 levels were associated with a slower rate of atrophy of entorhinal cortex (coefficient [95% CIs] = 0.040 [0.022, 0.058], *p* < 0.001; Figure 2B). In the model with middle temporal cortex as the dependent variable, the interaction term between plasma BMP6 and years was significant, indicating that higher baseline plasma BMP6 levels were associated with a slower rate of atrophy of middle temporal gyrus (coefficient [95% CIs] = 0.088 [0.040, 0.136], *p* < 0.001; Figure 2C). However, plasma BMP6 levels were not associated with the rate of atrophy of fusiform (coefficient [95% CIs] = −0.011 [−0.052, 0.030], *p* = 0.596; Figure 2D) or the enlargement of ventricles (coefficient [95% CIs] = −0.016 [−0.208, 0.176], *p* = 0.871; Figure 2E). In the model with whole brain as the dependent variable, the interaction term between plasma BMP6 and years was significant, indicating that higher baseline plasma BMP6 levels were associated with a slower rate of atrophy of whole brain (coefficient [95% CIs] = 2.656 [1.411, 3.900], *p* < 0.001; Figure 2F).

**Table 2:** Associations of plasma BMP6 with the rates of regional brain atrophy.

	**Hippocampus**		**Entorhinal cortex**		**Middle temporal gyrus**	
**Predictors**	**Coefficients [95% CIs]**	***P*** **-values**	**Coefficients [95% CIs]**	***P*** **-values**	**Coefficients [95% CIs]**	***P*** **-values**
Age	−0.026 [−0.036, −0.016]	<0.001	−0.008 [−0.015, −0.002]	0.014	−0.023 [−0.047, 0.001]	0.064
Years	−0.103 [−0.189, −0.018]	0.018	−0.026 [−0.151, 0.099]	0.684	−0.340 [−0.667, −0.014]	0.041
Female gender	0.167 [0.022, 0.313]	0.024	0.069 [−0.029, 0.167]	0.166	0.171 [−0.190, 0.532]	0.351
Education	−0.031 [−0.055, −0.007]	0.010	0.000 [−0.016, 0.016]	0.967	−0.037 [−0.096, 0.021]	0.211
APOE4 carriers	−0.342 [−0.484, −0.201]	<0.001	−0.237 [−0.331, −0.142]	<0.001	−0.138 [−0.488, 0.213]	0.441
MMSE	0.104 [0.064, 0.143]	<0.001	0.061 [0.034, 0.087]	<0.001	0.250 [0.152, 0.348]	<0.001
Plasma BMP6	0.138 [−0.086, 0.361]	0.227	0.148 [−0.002, 0.299]	0.052	0.073 [−0.483, 0.628]	0.797
Age × Years	−0.001 [−0.002, −0.001]	<0.001	−0.002 [−0.002, −0.001]	<0.001	−0.003 [−0.005, 0.000]	0.028
Female gender × Years	−0.035 [−0.044, −0.027]	<0.001	−0.032 [−0.044, −0.020]	<0.001	−0.114 [−0.145, −0.083]	<0.001
Education × Years	−0.003 [−0.004, −0.001]	<0.001	−0.005 [−0.006, −0.003]	<0.001	−0.012 [−0.017, −0.007]	<0.001
APOE4 carriers × Years	−0.065 [−0.073, −0.057]	<0.001	−0.039 [−0.051, −0.027]	<0.001	−0.205 [−0.236, −0.174]	<0.001
MMSE × Years	0.007 [0.004, 0.010]	<0.001	0.007 [0.003, 0.010]	0.001	0.023 [0.013, 0.033]	<0.001
Plasma BMP6 × Years	0.029 [0.016, 0.041]	<0.001	0.040 [0.022, 0.058]	<0.001	0.088 [0.040, 0.136]	<0.001
	**Fusiform**		**Ventricles**		**Whole brain**	
**Predictors**	**Coefficients [95% CIs]**	***P*** **-values**	**Coefficients [95% CIs]**	***P*** **-values**	**Coefficients [95% CIs]**	***P*** **-values**
Age	−0.032 [−0.052, −0.012]	0.002	0.600 [0.403, 0.798]	<0.001	−2.112 [-2.698, -1.526]	<0.001
Years	−0.588 [−0.870, −0.306]	<0.001	4.613 [3.314, 5.913]	<0.001	−14.067 [-22.604, -5.531]	0.001
Female gender	0.121 [−0.176, 0.417]	0.424	−5.584 [-8.511, -2.656]	<0.001	10.219 [1.531, 18.907]	0.021
Education	−0.036 [−0.084, 0.012]	0.145	0.190 [−0.286, 0.666]	0.433	−1.065 [-2.477, 0.348]	0.139
APOE4 carriers	−0.063 [−0.351, 0.225]	0.668	1.888 [−0.956, 4.732]	0.192	−7.614 [-16.056, 0.827]	0.077
MMSE	0.207 [0.127, 0.287]	<0.001	−0.983 [-1.773, −0.192]	0.015	3.133 [0.782, 5.484]	0.009
Plasma BMP6	0.132 [−0.324, 0.588]	0.570	−2.055 [-6.564, 2.454]	0.371	5.929 [-7.439, 19.298]	0.384
Age × Years	−0.003 [−0.005, −0.001]	0.008	0.003 [−0.007, 0.012]	0.554	−0.061 [−0.121, 0.000]	0.050
Female gender × Years	−0.048 [−0.075, −0.021]	<0.001	0.227 [0.102, 0.351]	<0.001	−1.898 [-2.718, -1.079]	<0.001
Education × Years	−0.008 [−0.012, −0.003]	<0.001	0.028 [0.009, 0.047]	0.004	−0.207 [−0.331, −0.083]	0.001
APOE4 carriers × Years	−0.149 [−0.176, −0.122]	<0.001	0.681 [0.556, 0.806]	<0.001	−4.216 [-5.033, -3.398]	<0.001
MMSE × Years	0.034 [0.025, 0.043]	<0.001	−0.142 [−0.183, −0.102]	<0.001	0.696 [0.427, 0.964]	<0.001
Plasma BMP6 × Years	−0.011 [−0.052, 0.030]	0.596	−0.016 [−0.208, 0.176]	0.871	2.656 [1.411, 3.900]	<0.001

Coefficients are unstandardized coefficients.

Cis, Confidence intervals; APOE, Apolipoprotein E; MMSE, Mini-Mental State Examination; BMP6, Bone morphogenetic protein 6.

### Supplementary analyses

First, apolipoprotein C-I (Apo C-I), which is also included in the multiplex-based immunoassay panel, is not a typical biomarker for cognitive decline or AD. Apo C-I, instead of BMP6, was used as the predictor of interest in the linear mixed-effects models. All other model specifications were kept the same as described in the Statistical analysis section. The Apo C-I × Years interaction term was not significant for all MRI features: hippocampus (coefficient [95% CIs] = −0.013 [−0.046, 0.020], *p* = 0.447), entorhinal cortex (coefficient [95% CIs] = −0.007 [−0.056, 0.042], *p* = 0.77), middle temporal cortex (coefficient [95% CIs] = −0.047 [−0.174, 0.081], *p* = 0.473), fusiform (coefficient [95% CIs] = 0.028 [−0.082, 0.137], *p* = 0.622), ventricles (coefficient [95% CIs] = 0.444 [−0.064, 0.952], *p* = 0.086), and whole brain (coefficient [95% CIs] = 2.403 [−0.902, 5.708], *p* = 0.154).

Second, we further examined the associations between plasma BMP6 levels and changes in the MMSE and Clinical Dementia Rating-Sum of Boxes (CDR-SB) scores over time. We found that higher baseline plasma BMP6 levels were associated with slower rates of changes in MMSE (BMP6 × Years term: coefficien*t* = 0.472, 95%CI = [0.294, 0.651], *p* < 0.001; Supplementary Table S1) and CDR-SB (BMP6 × Years term: coefficient = −0.307, 95%CI = [−0.415, −0.199], *p* < 0.001; Supplementary Table S2) over time.

Third, the relationships between plasma BMP6 levels and changes in six MRI features over times were investigated solely among MCI individuals. The overall pattern of the findings did not change. The results of the linear mixed-effects models for the hippocampus, entorhinal cortex, fusiform gyrus, middle temporal gyrus, ventricles, and whole brain are summarized in Supplementary Tables S3– S8, respectively.

Fourth, Spearman's correlation tests were performed to examine the cross-sectional relationships between plasma BMP6 and CSF biomarkers, including Aβ42, *t*-tau, and *p*-tau, in the overall sample. No significant relationship between plasma BMP6 and CSF Aβ42 was observed (rho = 0.07, *p* = 0.3). However, there were significant negative associations between plasma BMP6 and both t-tau (rho = −0.15, *p* = 0.038) and p-tau (rho = −0.16, *p* = 0.027) levels.

Fifth, MRI images from two timepoints (baseline and 5-year follow-up) for two MCI subjects with differing baseline plasma BMP6 levels are shown in Supplementary Figures S1 and S2.

## Discussion

This study examined the association between plasma BMP6 levels and the rates of regional brain atrophy among older adults without dementia. We found that higher levels of plasma BMP6 were associated with a slower rate of volume reduction in the hippocampus, entorhinal cortex, middle temporal gyrus, and whole brain. However, plasma BMP6 levels were not associated with changes in the volumes of the fusiform gyrus or ventricles. Our findings may provide novel insights into the mechanisms of neurodegeneration in AD, contributing to new avenues for timely intervention and potentially slowing disease progression.

The hippocampus, entorhinal cortex, and middle temporal gyrus are among the earliest and most severely affected regions in AD, with atrophy in these areas being a hallmark of the disease (16, 17). Our finding that higher plasma BMP6 levels are associated with slower atrophy in these brain regions is novel and suggests a potential neuroprotective effect of BMP6 (7). This finding is consistent with previous studies showing that BMP6 may play a crucial role in neuronal survival and synaptic plasticity (7), which are critical for maintaining cognitive performance. The supplementary analyses suggested that higher plasma BMP6 levels were associated with lower levels of CSF tau proteins. This finding may indicate that the potential neuroprotective effect of BMP6 on these brain regions is due to its ability to reduce tau pathologies, as tau pathology typically begins in these areas. Additionally, according to a recent study that tracked participants over time, higher blood BMP6 levels in older adults are associated with greater cognitive performance during the study's follow-up (9). However, few studies have investigated the relationship between plasma BMP6 levels and brain atrophy in living humans. Our study extends these findings by suggesting a potentially protective effect of BMP6 in specific brain regions associated with AD in older adults without dementia. Identifying blood biomarkers that predict the rate of brain atrophy is crucial for timely intervention and disease management. If confirmed in larger cohorts, plasma BMP6 levels could serve as a non-invasive tool to monitor disease progression and assess the efficacy of potential therapeutics. Additionally, therapeutic strategies targeting BMP6 signaling pathways may offer new avenues for the treatment of AD. For instance, pharmacological agents that increase BMP6 activity or mimic its effects could potentially slow down the rate of brain atrophy and neurodegeneration. Further studies are needed to explore the feasibility and safety of such approaches.

The lack of correlation between plasma BMP6 levels and alterations in the volumes of the fusiform gyrus or ventricles is intriguing. The fusiform gyrus is implicated in a variety of cognitive functions, including object naming performance (18). This region is typically not one of the earliest brain regions affected in AD (17). The enlargement of ventricles is a well-documented consequence of brain atrophy (19). However, the lack of association between BMP6 levels and ventricle size suggests that the influence of BMP6 is likely targeted to specific regions rather than being a broad indicator of brain atrophy.

Several limitations of this study should be considered. First, the sample size was relatively small, which may limit the generalizability of our findings. Future studies with larger and more diverse populations are needed to validate our findings. Second, association of CSF BMP6 levels and brain atrophy remains to be fully elucidated. Future studies are warranted to explore this research question. Finally, the observational nature of our study precludes causal inferences; further interventional studies are necessary to establish a causal relationship between BMP6 levels and brain atrophy.

In conclusion, our study provides evidence that higher plasma BMP6 levels are associated with a slower rate of volume reduction in the hippocampus, entorhinal cortex, middle temporal cortex, and whole brain among older adults without dementia. These findings offer novel insights into the mechanisms of neurodegeneration in AD and highlight the potential of BMP6 as a biomarker and therapeutic target.

## Data Availability

Publicly available datasets were analyzed in this study. The data used in the present study has been made publicly available by the Alzheimer's Disease Neuroimaging Initiative (ADNI) database (adni.loni.usc.edu).

## References

[1] Jack Jr CR Knopman DS Jagust WJ Shaw LM Aisen PS Weiner MW. Hypothetical model of dynamic biomarkers of the Alzheimer's pathological cascade. Lancet Neurol. (2010) 9:119–28. 10.1016/S1474-4422(09)70299-6 20083042 PMC2819840

[2] Querfurth HW Laferla FM. Alzheimer's disease. N Engl J Med. (2010) 362:329–44. 10.1056/NEJMra0909142 20107219

[3] De Toledo-Morrell L Goncharova I Dickerson B Wilson RS Bennett DA. From healthy aging to early Alzheimer's disease: in vivo detection of entorhinal cortex atrophy. Ann N Y Acad Sci. (2000) 911:240–53. 10.1111/j.1749-6632.2000.tb06730.x 10911878

[4] Devanand DP Pradhaban G Liu X Khandji A De Santi S Segal MH. Hippocampal and entorhinal atrophy in mild cognitive impairment: prediction of Alzheimer disease. Neurology. (2007) 68:828–36. 10.1212/01.wnl.0000256697.20968.d7 17353470

[5] Blennow K Zetterberg H. Biomarkers for Alzheimer's disease: current status and prospects for the future. J Intern Med. (2018) 284:643–63. 10.1111/joim.12816 30051512

[6] Peretti DE Boccalini C Ribaldi F Scheffler M Marizzoni M Ashton NJ. *Association of glial fibrillary acid* protein, Alzheimer's disease pathology and cognitive decline. Brain. (2024) 147:4094–04. 10.1093/brain/awae211 38940331 PMC11629700

[7] Sun L Guo C Wang T Li X Li G Luo Y. LIMK1 is involved in the protective effects of bone morphogenetic protein 6 against amyloid-β-induced neurotoxicity in rat hippocampal neurons. J Alzheimers Dis. (2014) 42:543–54. 10.3233/JAD-140231 24903778

[8] Crews L Adame A Patrick C Delaney A Pham E Rockenstein E. Increased BMP6 levels in the brains of Alzheimer's disease patients and APP transgenic mice are accompanied by impaired neurogenesis. J Neurosci. (2010) 30:12252–62. 10.1523/JNEUROSCI.1305-10.2010 20844121 PMC2978735

[9] Sun L Guo C Song Y Sheng J Xiao S. Blood BMP6 Associated with Cognitive Performance and Alzheimer's Disease Diagnosis: A Longitudinal Study of Elders. J Alzheimers Dis. (2022) 88:641–51. 10.3233/JAD-220279 35694925

[10] Petersen RC Aisen PS Beckett LA Donohue MC Gamst AC Harvey DJ. Alzheimer's Disease Neuroimaging Initiative (ADNI): clinical characterization. Neurology. (2010) 74:201–9. 10.1212/WNL.0b013e3181cb3e25 20042704 PMC2809036

[11] Folstein MF Folstein SE Mchugh PR. “Mini-mental state”. A practical method for grading the cognitive state of patients for the clinician. J Psychiatr Res. (1975) 12:189–98. 10.1016/0022-3956(75)90026-6 1202204

[12] Morris JC. The Clinical Dementia Rating (CDR): current version and scoring rules. Neurology. (1993) 43:2412–4. 10.1212/WNL.43.11.2412-a 8232972

[13] Nazeri A Ganjgahi H Roostaei T Nichols T Zarei M. Imaging proteomics for diagnosis, monitoring and prediction of Alzheimer's disease. Neuroimage 102 Pt 2. (2014) 657–65. 10.1016/j.neuroimage.2014.08.041 25173418 PMC6581536

[14] Arani A Borowski B Felmlee J Reid RI Thomas DL Gunter JL. Design and validation of the ADNI MR protocol. Alzheimers Dement. (2024) 20:6615–21. 10.1002/alz.14162 39115941 PMC11497751

[15] Fischl BR Salat DH Busa E Albert MS Dieterich M Haselgrove C. Whole brain segmentation automated labeling of neuroanatomical structures in the human brain. Neuron. (2002) 33:341–55. 10.1016/S0896-6273(02)00569-X 11832223

[16] Jack Jr CR Petersen RC O'brien PC Tangalos EG. MR-based hippocampal volumetry in the diagnosis of Alzheimer's disease. Neurology. (1992) 42:183–8. 10.1212/WNL.42.1.183 1734300

[17] Planche V Manjon JV Mansencal B Lanuza E Tourdias T Catheline G. Structural progression of Alzheimer's disease over decades: the MRI staging scheme. Brain Commun. (2022) 4:fcac109. 10.1093/braincomms/fcac109 35592489 PMC9113086

[18] Hays Weeks CC Zlatar ZZ Meloy MJ Shin DD Thomas L Wierenga CE. APOE genotype modifies the association of fusiform gyrus cerebral metabolic rate of oxygen consumption and object naming performance. J Alzheimers Dis. (2023) 91:1371–83. 10.3233/JAD-220749 36641668

[19] Nestor SM Rupsingh R Borrie M Smith M Accomazzi V Wells JL. Ventricular enlargement as a possible measure of Alzheimer's disease progression validated using the Alzheimer's disease neuroimaging initiative database. Brain. (2008) 131:2443–54. 10.1093/brain/awn146 18669512 PMC2724905

