# Supplementary material for: Association of plasma BMP6 levels with the rates of brain atrophy in older people without dementia
Source: Front Neurol. 2025 Jul 15;16:1559219. doi: 10.3389/fneur.2025.1559219 (PMC12303975; doi:10.3389/fneur.2025.1559219)
Supplement: Supplementary file 1 [file Table_1.docx]

**Table S1. Summary of linear mixed-effects model with MMSE as the outcome variable**

| Predictors | Coefficients [95%CIs] | P values |
| --- | --- | --- |
| Age | -0.011 [-0.055, 0.032] | 0.606 |
| Years | 0.310 [-0.486, 1.106] | 0.445 |
| Female gender | -0.001 [-0.642, 0.640] | 0.998 |
| Education | 0.117 [0.015, 0.219] | 0.025 |
| APOE4 carriers | -0.984 [-1.593, -0.376] | 0.002 |
| Plasma BMP6 | 0.555 [-0.424, 1.534] | 0.266 |
| Age × Years | -0.008 [-0.017, 0.001] | 0.083 |
| Female gender × Years | -0.218 [-0.339, -0.098] | <0.001 |
| Education × Years | -0.018 [-0.035, 0.000] | 0.052 |
| APOE4 carriers × Years | -0.603 [-0.718, -0.488] | <0.001 |
| Plasma BMP6 × Years | 0.472 [0.294, 0.651] | <0.001 |

**Table S2. Summary of linear mixed-effects model with CDR-SB as the outcome variable**

| Predictors | Coefficients [95%CIs] | P values |
| --- | --- | --- |
| Age | -0.007 [-0.032, 0.019] | 0.607 |
| Years | -0.534 [-1.022, -0.046] | 0.032 |
| Female gender | -0.159 [-0.540, 0.222] | 0.413 |
| Education | -0.019 [-0.080, 0.041] | 0.531 |
| APOE4 carriers | 0.432 [0.070, 0.794] | 0.019 |
| Plasma BMP6 | -0.226 [-0.808, 0.356] | 0.446 |
| Age × Years | 0.008 [0.003, 0.014] | 0.003 |
| Female gender × Years | 0.176 [0.103, 0.249] | <0.001 |
| Education × Years | 0.017 [0.006, 0.028] | 0.002 |
| APOE4 carriers × Years | 0.523 [0.453, 0.593] | <0.001 |
| Plasma BMP6 × Years | -0.307 [-0.415, -0.199] | <0.001 |

**Table S3. Summary of linear mixed-effects model with hippocampus as the outcome variable within MCI subjects**

| Predictors | Coefficients [95%CIs] | P values |
| --- | --- | --- |
| Age | -0.025 [-0.035, -0.015] | <0.001 |
| Years | -0.128 [-0.240, -0.015] | 0.026 |
| Female gender | 0.128 [-0.028, 0.284] | 0.106 |
| Education | -0.020 [-0.044, 0.005] | 0.117 |
| APOE4 carriers | -0.235 [-0.383, -0.087] | 0.002 |
| MMSE | 0.071 [0.029, 0.113] | <0.001 |
| Plasma BMP6 | 0.057 [-0.171, 0.285] | 0.623 |
| Age × Years | -0.001 [-0.002, 0.000] | 0.001 |
| Female gender × Years | -0.030 [-0.041, -0.019] | <0.001 |
| Education × Years | -0.002 [-0.004, 0.000] | 0.011 |
| APOE4 carriers × Years | -0.064 [-0.074, -0.055] | <0.001 |
| MMSE × Years | 0.007 [0.003, 0.010] | <0.001 |
| Plasma BMP6 × Years | 0.029 [0.015, 0.043] | <0.001 |

**Table S4. Summary of linear mixed-effects model with entorhinal cortex as the outcome variable within MCI subjects**

| Predictors | Coefficients [95%CIs] | P values |
| --- | --- | --- |
| Age | -0.009 [-0.016, -0.002] | 0.011 |
| Years | 0.025 [-0.140, 0.190] | 0.763 |
| Female gender | 0.050 [-0.057, 0.158] | 0.356 |
| Education | 0.007 [-0.010, 0.024] | 0.402 |
| APOE4 carriers | -0.190 [-0.292, -0.088] | <0.001 |
| MMSE | 0.046 [0.017, 0.074] | 0.002 |
| Plasma BMP6 | 0.081 [-0.075, 0.237] | 0.309 |
| Age × Years | -0.002 [-0.003, -0.001] | <0.001 |
| Female gender × Years | -0.032 [-0.047, -0.016] | <0.001 |
| Education × Years | -0.005 [-0.008, -0.003] | <0.001 |
| APOE4 carriers × Years | -0.033 [-0.046, -0.019] | <0.001 |
| MMSE × Years | 0.006 [0.001, 0.010] | 0.023 |
| Plasma BMP6 × Years | 0.040 [0.019, 0.060] | <0.001 |

**Table S5. Summary of linear mixed-effects model with fusiform gyrus as the outcome variable within MCI subjects**

| Predictors | Coefficients [95%CIs] | P values |
| --- | --- | --- |
| Age | -0.021 [-0.048, 0.006] | 0.123 |
| Years | -0.742 [-1.158, -0.325] | <0.001 |
| Female gender | 0.142 [-0.270, 0.554] | 0.499 |
| Education | -0.038 [-0.103, 0.027] | 0.246 |
| APOE4 carriers | -0.027 [-0.418, 0.365] | 0.893 |
| MMSE | 0.212 [0.102, 0.323] | <0.001 |
| Plasma BMP6 | -0.054 [-0.654, 0.547] | 0.861 |
| Age × Years | -0.002 [-0.004, 0.001] | 0.208 |
| Female gender × Years | -0.066 [-0.106, -0.027] | 0.001 |
| Education × Years | -0.006 [-0.012, 0.000] | 0.056 |
| APOE4 carriers × Years | -0.208 [-0.242, -0.173] | <0.001 |
| MMSE × Years | 0.030 [0.018, 0.042] | <0.001 |
| Plasma BMP6 × Years | 0.094 [0.041, 0.146] | <0.001 |

**Table S6. Summary of linear mixed-effects model with middle temporal gyrus as the outcome variable within MCI subjects**

| Predictors | Coefficients [95%CIs] | P values |
| --- | --- | --- |
| Age | -0.022 [-0.043, -0.001] | 0.042 |
| Years | -0.550 [-0.910, -0.191] | 0.003 |
| Female gender | 0.207 [-0.116, 0.531] | 0.209 |
| Education | -0.022 [-0.073, 0.029] | 0.395 |
| APOE4 carriers | -0.020 [-0.328, 0.288] | 0.898 |
| MMSE | 0.190 [0.103, 0.277] | <0.001 |
| Plasma BMP6 | -0.066 [-0.538, 0.406] | 0.784 |
| Age × Years | -0.004 [-0.006, -0.001] | 0.002 |
| Female gender × Years | -0.050 [-0.084, -0.015] | 0.005 |
| Education × Years | -0.007 [-0.013, -0.002] | 0.005 |
| APOE4 carriers × Years | -0.129 [-0.159, -0.099] | <0.001 |
| MMSE × Years | 0.033 [0.023, 0.044] | <0.001 |
| Plasma BMP6 × Years | -0.001 [-0.047, 0.044] | 0.959 |

**Table S7. Summary of linear mixed-effects model with ventricles as the outcome variable within MCI subjects**

| Predictors | Coefficients [95%CIs] | P values |
| --- | --- | --- |
| Age | 0.620 [0.404, 0.835] | <0.001 |
| Years | 4.567 [2.838, 6.295] | <0.001 |
| Female gender | -5.086 [-8.399, -1.774] | 0.003 |
| Education | 0.117 [-0.404, 0.638] | 0.659 |
| APOE4 carriers | 0.971 [-2.179, 4.120] | 0.545 |
| MMSE | -0.626 [-1.516, 0.265] | 0.168 |
| Plasma BMP6 | -1.529 [-6.368, 3.310] | 0.535 |
| Age × Years | 0.001 [-0.010, 0.012] | 0.881 |
| Female gender × Years | 0.329 [0.163, 0.496] | <0.001 |
| Education × Years | 0.032 [0.007, 0.058] | 0.014 |
| APOE4 carriers × Years | 0.740 [0.593, 0.887] | <0.001 |
| MMSE × Years | -0.134 [-0.185, -0.084] | <0.001 |
| Plasma BMP6 × Years | -0.099 [-0.320, 0.123] | 0.381 |

**Table S8. Summary of linear mixed-effects model with whole brain as the outcome variable within MCI subjects**

| Predictors | Coefficients [95%CIs] | P values |
| --- | --- | --- |
| Age | -2.129 [-2.746, -1.513] | <0.001 |
| Years | -22.761 [-33.630, -11.893] | <0.001 |
| Female gender | 5.850 [-3.630, 15.329] | 0.226 |
| Education | -0.727 [-2.218, 0.764] | 0.338 |
| APOE4 carriers | -5.032 [-14.037, 3.974] | 0.272 |
| MMSE | 1.755 [-0.796, 4.306] | 0.177 |
| Plasma BMP6 | 1.249 [-12.567, 15.064] | 0.859 |
| Age × Years | -0.050 [-0.120, 0.020] | 0.164 |
| Female gender × Years | -1.019 [-2.063, 0.026] | 0.056 |
| Education × Years | -0.058 [-0.217, 0.101] | 0.472 |
| APOE4 carriers × Years | -3.862 [-4.771, -2.953] | <0.001 |
| MMSE × Years | 0.843 [0.528, 1.158] | <0.001 |
| Plasma BMP6 × Years | 2.970 [1.603, 4.336] | <0.001 |


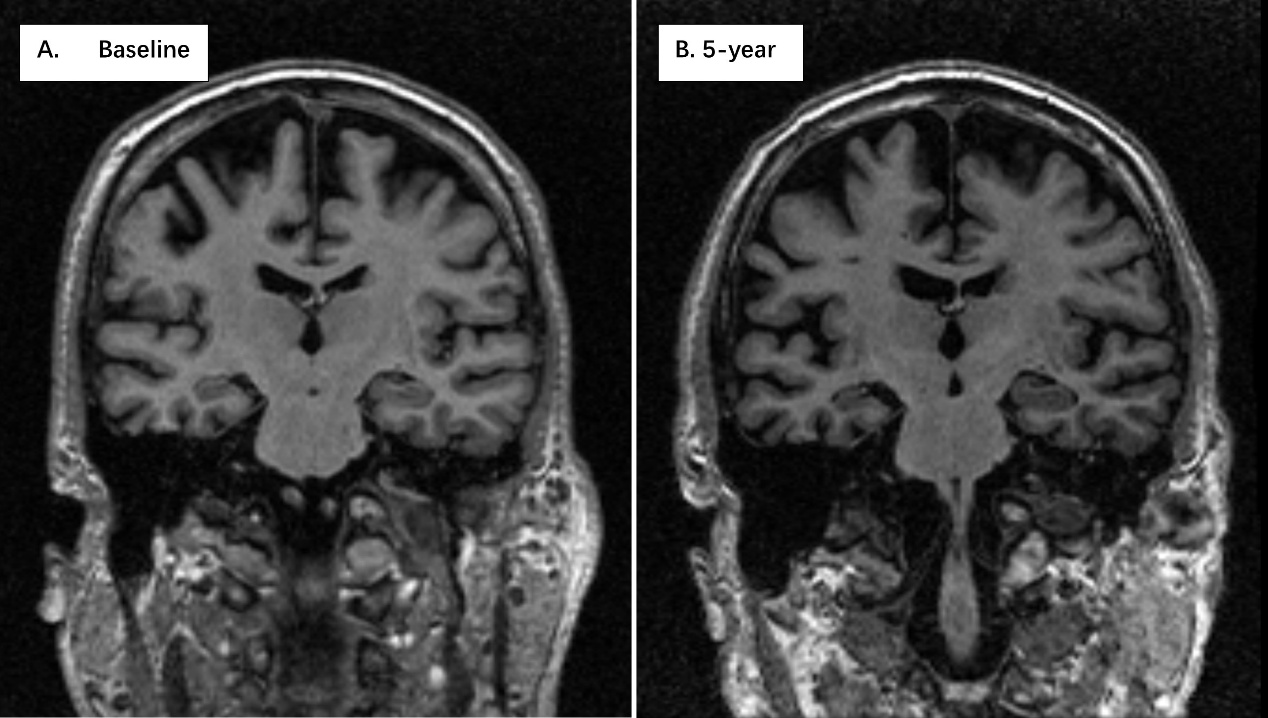


**Figure S1. Baseline and 5-year T1 MRI images from an MCI subject with a relatively high baseline plasma BMP6 level (1.15 ng/mL).**


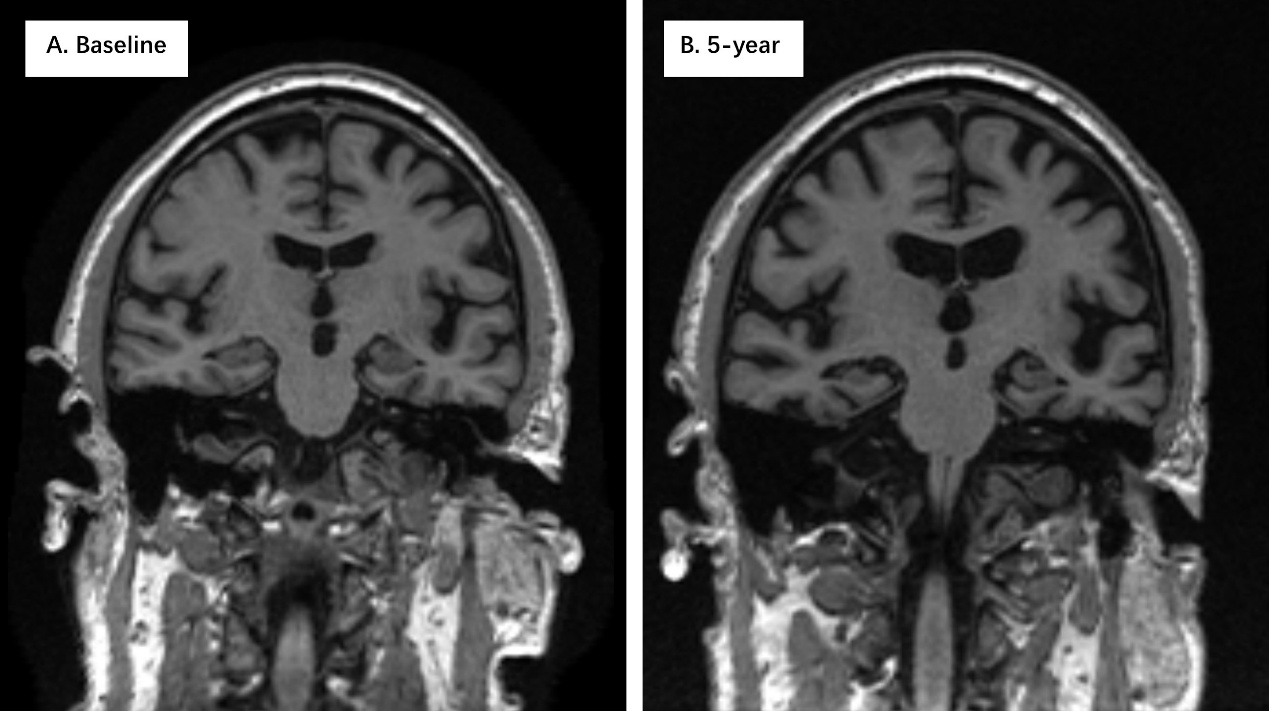


**Figure S2. Baseline and 5-year T1 MRI images from an MCI subject with a relatively low baseline plasma BMP6 level (0.84 ng/mL).**
